# Clinical responses to EGFR-tyrosine kinase inhibitor retreatment in non-small cell lung cancer patients who benefited from prior effective gefitinib therapy: a retrospective analysis

**DOI:** 10.1186/1471-2407-11-1

**Published:** 2011-01-01

**Authors:** Satoshi Watanabe, Junta Tanaka, Takeshi Ota, Rie Kondo, Hiroshi Tanaka, Hiroshi Kagamu, Kosuke Ichikawa, Jun Koshio, Junko Baba, Takao Miyabayashi, Ichiei Narita, Hirohisa Yoshizawa

**Affiliations:** 1 Department of Medicine (II), Niigata University Medical and Dental Hospital, Niigata City, Niigata, Japan; 2 Bioscience Medical Research Center, Niigata University Medical and Dental Hospital, Niigata City, Niigata, Japan

## Abstract

**Background:**

Gefitinib was the first epidermal growth factor receptor-tyrosine kinase inhibitor (EGFR-TKI) approved for the treatment of advanced non-small cell lung cancer (NSCLC). Few treatment options are available for NSCLC patients who have responded to gefitinib treatment and demonstrated tumor progression. The present study was conducted to evaluate the efficacy and toxicity of the 2^nd ^EGFR-TKI administration.

**Methods:**

We retrospectively analyzed 11 patients who had obtained a partial response (PR) or stable disease (SD) with gefitinib treatment and were re-treated with EGFR-TKI after failure of the initial gefitinib treatment.

**Results:**

Three patients (27%) were treated with gefitinib as the 2^nd ^EGFR-TKI, and 8 patients (73%) received erlotinib. Only one patient (9%) showed PR, 7 (64%) achieved SD, and 3 (27%) had progressive disease. The disease control rate was 73% (95% CI, 43% - 91%) and the median progression-free survival was 3.4 months (95% CI, 2 - 5.2). The median overall survival from the beginning of the 2^nd ^EGFR-TKI and from diagnosis were 7.3 months (95% CI, 2.7 - 13) and 36.7 months (95% CI, 23.6 - 43.9), respectively. No statistical differences in PFS or OS were observed between gefitinib and erlotinib as the 2^nd ^EGFR-TKI (PFS, P = 0.23 and OS, P = 0.052). The toxicities associated with the 2^nd ^EGFR-TKI were generally acceptable and comparable to those observed for the initial gefitinib therapy.

**Conclusions:**

Our results indicate that a 2^nd ^EGFR-TKI treatment can be an effective treatment option for gefitinib responders.

## Background

Gefitinib was the first epidermal growth factor receptor tyrosine kinase inhibitor (EGFR-TKI) to become available for the treatment of non-small cell lung cancer (NSCLC). Several studies have demonstrated that gefitinib is effective for the second-line treatment of NSCLC [1-3]. Although the phase III ISEL trial failed to prove the superiority of gefitinib treatment compared to placebo in previously treated patients, a subgroup analysis demonstrated improved survival in particular populations (Asians and non-smokers) [4]. Further analyses in other studies have also revealed that clinical factors (Asians, females, non-smokers, and adenocarcinoma histology) are associated with the response to gefitinib treatment [5]. EGFR mutations, such as the deletion of exon 19 and the single L858R mutation in exon 21, have also been reported to be correlated with a longer survival and were found more frequently in Asian patients [6-8]. Recently, a superior progression-free survival (PFS) with gefitinib compared with the combination of carboplatin and paclitaxel in untreated NSCLC patients with predictors of gefitinib sensitivity was proven in two large phase III studies [9,10]. Gefitinib is now recommended for advanced or metastatic NSCLC patients under such circumstances as a first or a second-line treatment.

Despite the high disease control rate (DCR), gefitinib treatment is not curative and eventually there is disease recurrence, even in patients with predictors of sensitivity. For the many NSCLC patients who previously responded to gefitinib but later showed tumor progression, very few treatment options are available.

Some investigators have conducted studies to evaluate the efficacy of EGFR-TKI re-administration [11-14]. In most of those studies, both gefitinib responders and non-responders were retreated with gefitinib or erlotinib, and gefitinib responders tended to benefit from the 2^nd ^EGFR-TKI.

Here, we retrospectively analyzed the efficacy of the 2^nd ^EGFR-TKI administration after failure of the initial gefitinib treatment in NSCLC patients who had previously achieved disease control with gefitinib. The risks of the 2^nd ^administration of EGFR-TKI, especially the association with adverse events in the initial gefitinib treatment, were also evaluated.

## Methods

### Patients

We conducted a retrospective search of the medical records at Niigata University Medical and Dental Hospital, from June 2005 through October 2009, and we identified 11 NSCLC patients who had obtained a partial response (PR) or stable disease (SD) with gefitinib treatment and undergone EGFR-TKI retreatment sometime after the failure of the initial gefitinib treatment. All patients were treated initially with oral gefitinib at a dose of 250 mg/day, which was continued until either a radiographic tumor or overt clinical progression was observed. The same dose of gefitinib, or erlotinib at a dose of 150 mg/day, was used for EGFR-TKI retreatment and continued until tumor progression was detected.

### Assessment of the response and adverse events

The tumor response was evaluated by radiologic examinations according to the Response Evaluation Criteria in Solid Tumors (RECIST) [15]. Disease control was defined as complete response (CR), PR or SD. PFS and overall survival (OS) were defined as the period from the start of the treatment to the date when disease progression and death, respectively, were observed.

Adverse events were assessed according to Common Terminology Criteria for Adverse Events of the National Cancer Institute (version 3.0) [16].

### Statistical analysis

PFS and OS estimates were obtained using the Kaplan-Meier method.

## Results

### Patient characteristics

Of the 11 identified patients who benefited from gefitinib and were retreated with EGFR-TKI, 3 patients (27%) received gefitinib and 8 patients (73%) received erlotinib as the 2^nd ^round of EGFR-TKI. As shown in Table 1 the ages of patients ranged from 46 to 70 years (median, 55 years), and there were 8 females (73%), 7 non-smokers (64%), and 10 adenocarcinoma patients (91%). Three patients (27%) exhibited EGFR gene mutations, but the mutation statuses of the other 8 patients (73%) were not determined. All patients had received platinum-based chemotherapy before the initial gefitinib treatment. The patient characteristics, including treatment backgrounds and responses, are summarized in Table 2.

**Table 1:** Patient Characteristics 1

Characteristics	No. of Patients	%
Total enrolled	11	
Gender		
Female	8	73
Male	3	27
Age (y)		
Median	55	
Range	46-70	
ECOG performance status		
1	6	55
2	0	0
3	3	27
4	2	18
Histology		
Adenocarcinoma	10	91
Squamous	1	9
Smoking history		
Current	3	27
Ex-smoker	1	9
Never	7	64
EGFR mutation		
Exon 19 deletion	2	18
L858R	1	9
Not available	8	73

EGFR, epidermal growth factor receptor.

**Table 2:** Patient Characteristics 2

Case	Age (y)	Gender	Smoking	Histology	EGFR mutation	PFS to 1^st ^TKI	TKI sequence	Interval from 1^st ^and 2^nd^	Chemo. after 1^st^	PS	Response	PFS to 2^nd ^TKI	OS from 2^nd ^TKI
1	50	F	Current	Ad	NA	9.8	G→E	7.9	CBDCA+GEM	1	PD	0.9	13.1
2	46	F	Never	Ad	NA	11.8	G→G	4.5	DOC	1	PR	6.4	24.6
3	58	F	Ex	Ad	19 deletion	38.4	G→G	2.8	DOC	1	SD	7.3	24.1
4	70	F	Never	Sq	NA	10.2	G→E	12.8	GEM	1	SD	1.7	4.3
5	60	F	Never	Ad	NA	13	G→G	5.4	GEM	1	PD	1.6	2.1
6	63	F	Never	Ad	NA	7.4	G→E	2.6	-	3	SD	3.6	7.8
7	52	M	Never	Ad	L858R	5.8	G→E	1	-	4	SD	6.4	6.4
8	51	M	Current	Ad	NA	4.3	G→E	1.6	AMR	3	PD	0.6	0.9
9	61	F	Never	Ad	NA	8.5	G→E	2.3	VNR	3	SD	2.9	4
10	53	F	Never	Ad	NA	12.9	G→E	0	-	4	SD	6.2	7.3
11	54	M	Current	Ad	19 deletion	3.8	G→E	7.3	VNR	1	SD	3.2	5

PFS, progression-free survival; TKI, tyrosine kinase inhibitor; PS, performance status; OS, overall survival; F, female; M, male; Ex, ex-smoker; Ad, adenocarcinoma; Sq, squamous cell carcinoma; G, gefitinib; E, erlotinib; CBDCA, carboplatin; GEM, gemcitabine; DOC, docetaxel; AMR, amrubicin; VNR, vinorelbine; PR, partial response; SD, stable disease; PD, progressive disease.

### Response to the initial gefitinib treatment

During the 1^st ^EGFR-TKI treatment with gefitinib, 8 patients achieved PR as the best response (73%, Table 3), and 3 patients (27%) were SD. The median PFS was 9.8 months, with a 95% CI of 6.6 to 16.7 months.

**Table 3:** Summary of prior therapy

Characteristics	No. of patients	%
No. of chemotherapy regimens before gefitinib		
1	2	18
2	4	36
3	4	36
4	1	9
Best response to gefitinib		
PR	8	73
SD	3	27
PFS to gefitinib		
Median	9.8	
95% CI	6.6 - 16.7	
Interval from discontinuation of gefitinib to 2^nd ^EGFR-TKI		
Median	2.8	
95% CI	1.9 - 6.9	

### Response to the 2^nd ^EGFR-TKI

Three patients (27%) received the 2^nd ^EGFR-TKI immediately after gefitinib failure, and 8 (73%) underwent 1 cytotoxic regimen between the initial gefitinib and the 2^nd ^EGFR-TKI treatments. The median interval from the discontinuation of gefitinib to the 2^nd ^EGFR-TKI was 2.8 months (95% CI, 1.9 - 6.9, Table 3). Only one patient (9%) demonstrated PR, 7 (64%) remained SD, and 3 (27%) had PD. The DCR was 73% (95% CI, 43% - 91%) and the median PFS was 3.4 months (95% CI, 2 - 5.2). The median OS from the beginning of the 2^nd ^EGFR-TKI and from diagnosis were 7.3 months (95% CI, 2.7 - 13.0) and 36.7 months (95% CI, 23.6 - 43.9), respectively. No statistical differences in PFS or OS were observed between gefitinib and erlotinib as the 2^nd ^EGFR-TKI (PFS, P = 0.23 and OS, P = 0.052).

In contrast with previous studies, we further compared the clinical courses of the patients with those of gefitinib responders who were not treated with a 2^nd ^EGFR-TKI following gefitinib failure. We reviewed the medical records at our institute and found 9 patients with backgrounds that were similar to those of the 2^nd ^EGFR-TKI patients (sex, age (< 70 years old or > 70 years old), histology, and response to gefitinib treatment). No statistical differences in PFS to 1^st ^gefitinib treatment were noted between both groups (9.8 months in the 2^nd ^TKI group and 8.7 months (95% CI, 7.6 - 9.8) in the control group, P = 0.87). All of the identified control patients had been treated with platinum-doublet chemotherapy before gefitinib but had not received 2^nd ^EGFR-TKI. The OS from the start of the initial gefitinib treatment tended to be longer in patients who received a 2^nd ^EGFR-TKI (median OS, 21.5 months (95% CI, 14.6 - 28.4)) compared to those in the control group (median OS, 12.3 months (95% CI, 9.4 - 15.2), P = 0.07).

In the control group, 5 out of 9 patients had been treated with cytotoxic chemotherapy after gefitinib failure. To compare the efficacy of the 2^nd ^EGFR-TKI with chemotherapy after disease progression with gefitinib, data were collected from these 5 patients in the control group who had received chemotherapy after gefitinib failure (Table 4). The DCR for chemotherapy after gefitinib treatment was 20% and comprised one SD and four PD. The median PFS and OS from the start of chemotherapy after gefitinib treatment were only 2 months (95% CI, 1.5 - 2.4) and 2.5 months (95% CI, 2.2 - 2.8), respectively. No significant differences in the PFS or OS from the start of treatment after gefitinib were observed between the patients who received a 2^nd ^EGFR-TKI and those who underwent cytotoxic chemotherapy (PFS, P = 0.1 and OS, P = 0.12); however, a 2^nd ^EGFR-TKI appeared to be a better option for gefitinib responders.

**Table 4:** Tumor response to 2nd EGFR-TKI vs. chemotherapy

Characteristics	2^nd ^TKI group	Control group	P
OS from 1^st ^gefitinib			
Median	21.5	12.3	0.07
95% CI	14.6 - 28.4	9.4 - 15.2	
Response to 2^nd ^TKI or chemotherapy			
PR	1	0	
SD	7	1	
PD	3	4	
PFS to 2^nd ^TKI or chemotherapy			
Median	3.4	2	0.1
95% CI	2 - 5.2	1.5 - 2.4	
OS from 2^nd ^TKI or chemotherapy			
Median	7.3	2.2	0.12
95% CI	2.7 - 13	2.2 - 2.8	

### Toxicity profiles for the initial gefitinib and 2^nd ^EGFR-TKI treatments

To determine whether the initial gefitinib treatment and EGFR-TKI retreatment caused similar adverse events, we assessed the toxicity profiles of all 11 patients (Table 5). The most common toxicity associated with both treatments was a grade 1/2 skin rash. Although one patient presented a grade 3 elevation of γ-glutamyltranspeptidase during both treatment with gefitinib and with erlotinib (patient no. 7), the other observed toxicities were generally acceptable. In 5 patients, the toxicity profiles for the initial gefitinib and the 2^nd ^EGFR-TKI treatments were similar. None of the patients demonstrated interstitial lung disease in response to EGFR-TKI.

**Table 5:** Toxicity profiles for the initial gefitinib and 2nd EGFR-TKI treatments. Adverse events were evaluated according to Common Terminology Criteria for Adverse Events of the National Cancer Institute (version 3.0).

Case	Initial gefitinib	2^nd ^EGFR-TKI
1	-	Rash G2
2	Rash G2	-
3	-	-
4	Rash G2, Liver G1, Diarrhea G2	Rash G1, Diarrhea G1
5	Rash G1	Rash G2
6	Diarrhea G2, Taste alteration G2	Rash G1, Diarrhea G1
7	Rash G2, Liver G3	Rash G2, Liver G3
8	Rash G2	Liver G2
9	Rash G1, Nail G1, Nausea G1	Rash G1
10	Liver G1	-
11	-	Rash G1, Diarrhea G1

G, grade; Liver, serum glutamic pyruvic transminase, serum glutamic oxaloacetic transaminase and γ-glutamyltranspeptidase.

## Discussion

To the best of our knowledge, 18 cases of patients who received gefitinib re-administration after failure of the initial gefitinib treatment have been reported to date, including 3 cases reported by our group (Table 6) [17-21]. All 18 patients responded to the initial gefitinib treatment, and most of the cases underwent cytotoxic chemotherapy between the first and second gefitinib therapy. Fourteen patients benefited from the 2^nd ^gefitinib treatment, and the overall DCR was 78%. In our 3 patients, the toxicity of the 2^nd ^gefitinib was similar to that observed for the initial gefitinib treatment and was acceptable. Gefitinib retreatment is likely a good option for patients who have demonstrated a response to a previous gefitinib treatment.

**Table 6:** Patient characteristics of the previous studies of gefitinib readministration

		Response to gefitinib		Response to 2^nd ^gefitinib	
			
Author	No. of patients	CR/PR/SD	PD	CR/PR/SD	PD
Yokouchi H et al.	9	9	-	8	1
Yoshimoto A et al.	1	1	-	1	0
Yano S et al.	3	3	-	2	1
Hashimoto N et al.	1	1	-	0	1
Kurata T et al.	1	1	-	1	0

CR, complete response.

Clinical studies have demonstrated that erlotinib is effective even in patients who are not considered to be good responders to gefitinib, such as those with a negative EGFR mutation, squamous cell carcinoma, or a history of smoking [22]. Because erlotinib is used at its maximum tolerated dose, whereas gefitinib is used at only about one-third of its maximum tolerated dose in daily practice, the biological activity of erlotinib at standard doses may be higher than that of gefitinib [2,4,23-25]. These reports suggest that erlotinib may be active even in patients who demonstrated tumor progression during a prior gefitinib treatment. Thus, erlotinib has been selected as a treatment option for use after gefitinib failure (Table 7) [11-14,26-33]. In most studies, including the present investigation, favorable results have been documented, and the authors have concluded that erlotinib appears to be a useful treatment after gefitinib failure.

**Table 7:** Patient characteristics of the previous studies of erlotinib after gefitinib failure

		Response to gefitinib		Response to erlotinib		
			
Author	No. of patients	CR/PR/SD	PD	CR/PR/SD	PD	DCR (%)
Lee DH et al.	23	17	6	2	21	9
Cho BC et al.	21	10	11	6	15	29
Viswanathan A et al.	5	4	1	0	5	0
Costa DB et al.	18	16	2	4	14	22
Sim SH et al.	16	11	5	4	12	25
Chang JW et al.	1	1	0	1	0	100
Garfield DH et al.	1	1	0	1	0	100
Vasile E et al.	8	8	0	5	3	63
Gridelli C et al.	3	3	0	3	0	100
Wong AS et al.	14	9	5	5	9	36
Zhou ZT et al.	21	15	6	10	11	48
Wong MK et al.	21	18	3	12	9	57

Although it is difficult to address the precise mechanism underlying these results, several studies have suggested a possible explanation for the clinical benefit of EGFR-TKI retreatment. Some cytotoxic agents have been reported to restore the sensitivity of NSCLC cells to gefitinib in vitro by increasing EGFR phosphorylation [34,35]. It is also possible that chemotherapy during the EGFR-TKI-free interval could decrease EGFR-TKI resistant tumor cells. However, no significant differences in PFS or OS were observed between our patients who received chemotherapy before the 2^nd ^EGFR-TKI and those who received the 2^nd ^EGFR-TKI immediately after gefitinib failure. In addition, the duration between the initial gefitinib and the 2^nd ^EGFR-TKI treatments was not associated with the response to 2^nd ^EGFR-TKI. Similarly to these findings, other researchers have found no evidence that either chemotherapy among the 1^st ^and 2^nd ^EGFR-TKIs or the duration of the EGFR-TKI-free period affects either PFS or OS in the 2^nd ^EGFR-TKI [31,33].

Secondary EGFR mutations might be associated with the efficacy of erlotinib after gefitinib failure. MET amplification and secondary EGFR mutations, such as T790 M, L747 S, D761Y, and T854A have been identified in NSCLC patients with an acquired resistance to EGFR-TKI [36-42]. T790 M mutation was found in 50%, MET amplification in 20%, and other secondary mutations in less than 5% of the NSCLC patients carrying EGFR mutations with TKI resistance [43,44]. In vitro studies have revealed that tumor cells carrying non-T790 M mutations show a partial resistance to EGFR-TKI, but are much less resistant compared to cells with T790 M. These data suggest that an increased EGFR-TKI dose might circumvent the acquired resistance caused by non-T790 M mutations. Previous studies have indicated that the serum concentration of erlotinib is several-fold higher than that of gefitinib at standard doses [24,25]. This difference in biological activities between the TKIs may contribute to the efficacy of erlotinib after gefitinib failure in patients carrying non-T790 M mutations.

In conclusion, our findings suggest that a 2^nd ^EGFR-TKI can be a treatment option for patients who benefited from a previous gefitinib treatment. However, as shown in Table 7 some studies failed to demonstrate the efficacy of 2^nd ^EGFR-TKI after gefitinib failure. Cho et al. mentioned that the tumor response to 1^st ^gefitinib treatment can be a predictive marker [14]. They described that patients who showed SD during 1^st ^gefitinib treatment had better survival with 2^nd ^EGFR-TKI, however those who had PD to 1^st ^gefitinib rarely responded to 2^nd ^EGFR-TKI. The difference in the percentage of patients with a good predictor might affect the results of these trials about 2^nd ^EGFR-TKI. Intense research has been devoted to clarifying the mechanism responsible for acquired resistance, but it is difficult to obtain clinical samples from all patients to confirm MET amplification or secondary mutations. Jackman et al. recently published a clinical definition of acquired resistance to EGFR-TKI [45]. This consensus definition will facilitate the establishment of standard entry criteria for studies investigating acquired resistance. All of our patients except one met these criteria (no. 8 in Table 2). Despite rapid tumor progression during a previous cytotoxic chemotherapy, this patient obtained SD with an initial gefitinib therapy of 4.3 months, and therefore we considered this patient to have benefited from the gefitinib treatment. Further clinical trials are required to develop a novel treatment for patients with acquired resistance.

## Conclusion

In the current study, we analyzed the efficacy and toxicity of a 2^nd ^EGFR-TKI treatment in patients who demonstrated a response to prior gefitinib therapy and tumor progression. A second EGFR-TKI treatment was generally effective in patients who had benefited from the initial gefitinib therapy. The adverse events associated with a 2^nd ^EGFR-TKI were acceptable and comparable with those observed for the initial gefitinib therapy. In Japan, gefitinib has been approved for the treatment of inoperable and recurrent NSCLC since 2002, and many patients have already experienced a need for a new treatment option following gefitinib treatment. Based on the present data, a 2^nd ^EGFR-TKI treatment could represent a potentially new treatment for gefitinib responders. Prospective clinical trials and translational analyses in this area of research are warranted.

## Competing interests

The authors declare that they have no competing interests.

## Authors' contributions

SW conducted the study and drafted the manuscript. JT conceived and designed the study and collected the clinical data. TO, RK, HT, HK and TM participated in the patient care, and collected the data. KI, JK and JB analyzed and interpreted the data. IN and HY provided the administrative support. All the authors have read and approved the final version of the manuscript.

## Pre-publication history

The pre-publication history for this paper can be accessed here:

http://www.biomedcentral.com/1471-2407/11/1/prepub
